# Obsessive-Compulsive Disorder and suicide: what do we know up until now?

**DOI:** 10.1192/j.eurpsy.2022.1660

**Published:** 2022-09-01

**Authors:** G. Salvadori, L. Osaku, M. Porcu

**Affiliations:** 1 Maringa State University, Medicine, Maringa, Brazil; 2 Maringa State University, Medicine, Maringá, Parana, Brasil, Brazil

**Keywords:** Suicide, obsessive-compulsive disorder, suicidal behavior, suicidal ideation

## Abstract

**Introduction:**

Obsessive-Compulsive Disorder (OCD) was recently (in 2015) associated with suicidal risk, regardless of depression or other mental disorders, but it is a disorder with great heterogeneity, and the subtypes involved in this risk have not yet been clarified.

**Objectives:**

To verify which patterns of OCD symptoms are more associated with suicidal risk, other possible risk factors and better treatment options evidenced in the literature.

**Methods:**

Literature review with predefined search criteria and keywords in electronic databases (Pubmed, Virtual Health Library and Cochrane) between 2015 and 2020. Identification, analysis, calculation and synthesis of the results were carried out in a descriptive and qualitative manner.

**Results:**

Twenty five articles were included. Obsessions of unacceptable thoughts pattern, perfectionism traits and alexithymia are important predictors of suicidal risk. Compulsions were not associated with suicide. Depression is the comorbidity with the greatest impact on suicidal ideation. Better socioeconomic status, education, and female gender are protective factors for mortality. The method chosen for attempts is preferably non-violent (drug intoxication). Treatments derived from cognitive behavioral therapy are currently being investigated further, and in addition to the evidence for the use of selective serotonin reuptake inhibitors, antipsychotics have been used as an adjuvant.

**Conclusions:**

Unacceptable thoughts play an important role in suicide resulting from OCD, and the absence of compulsive behavior may be correlated to suicide risk. Treatment directed at cognitions seems relevant. Further studies are needed to clarify the appropriate approach in this subtype of the disease.

**Disclosure:**

No significant relationships.

